# The impact of date palm fruits and their component polyphenols, on gut microbial ecology, bacterial metabolites and colon cancer cell proliferation

**DOI:** 10.1017/jns.2014.16

**Published:** 2014-10-08

**Authors:** Noura Eid, Sumia Enani, Gemma Walton, Giulia Corona, Adele Costabile, Glenn Gibson, Ian Rowland, Jeremy P. E. Spencer

**Affiliations:** Department of Food and Nutritional Sciences, School of Chemistry, Food and Pharmacy, University of Reading, Reading RG6 6AP, UK

**Keywords:** Date palm fruit, Date polyphenols, Gut ecology, Colonic cancer inhibition, DDE, digested date extract, DPE, date polyphenol extract, FISH, fluorescence *in situ* hybridisation, FOS, fructo-oligosaccharide, SRB, sulforhodamine B

## Abstract

The fruit of the date palm (*Phoenix dactylifera* L.) is a rich source of dietary fibre and polyphenols. We have investigated gut bacterial changes induced by the whole date fruit extract (digested date extract; DDE) and its polyphenol-rich extract (date polyphenol extract; DPE) using faecal, pH-controlled, mixed batch cultures mimicking the distal part of the human large intestine, and utilising an array of microbial group-specific 16S rRNA oligonucleotide probes. Fluorescence microscopic enumeration indicated that there was a significant increase in the growth of bifidobacteria in response to both treatments, whilst whole dates also increased bacteroides at 24 h and the total bacterial counts at later fermentation time points when compared with DPE alone. Bacterial metabolism of whole date fruit led to the production of SCFA, with acetate significantly increasing following bacterial incubation with DDE. In addition, the production of flavonoid aglycones (myricetin, luteolin, quercetin and apigenin) and the anthocyanidin petunidin in less than 1 h was also observed. Lastly, the potential of DDE, DPE and metabolites to inhibit Caco-2 cell growth was investigated, indicating that both were capable of potentially acting as antiproliferative agents *in vitro*, following a 48 h exposure. This potential to inhibit growth was reduced following fermentation. Together these data suggest that consumption of date fruits may enhance colon health by increasing beneficial bacterial growth and inhibiting the proliferation of colon cancer cells. This is an early suggestion that date intake by humans may aid in the maintenance of bowel health and even the reduction of colorectal cancer development.

The human colon harbours about 10^13^ micro-organisms and over 1000 species of bacteria and is considered the most metabolically active site in the human body^(^^1^^)^. These intestinal microbiota have been shown to play a prominent role in maintaining gut health and affecting the aetiology and pathogenesis of a wide range of disease and disorders, including inflammatory bowel disease, diarrhoea and colorectal cancer^(^^2^^)^. Identifying dietary regimens and components that can beneficially modify the gut microbiota represents a possible strategy for reducing the risk of the development of such diseases^(^^3^^)^. Much research in this regard has focused on ‘prebiotics’, and in particular the ability of certain types of dietary fibre, especially indigestible oligosaccharides, to stimulate the growth of and/or activity of beneficial gut bacteria such as bifidobacteria and lactobacilli leading to a concomitant positive effect on colonic health^(^^3^^,^^4^^)^. Recent work has focused on prebiotics and other fibre-rich foods that may be fermented by the lower gut, and insoluble compounds^(^^5^^–^^11^^)^ and polyphenols^(^^12^^–^^17^^)^ that modify the gut ecology and increase beneficial types of bacteria. Indeed, a large number of human intervention studies have indicated that foods rich in fibres^(^^18^^–^^25^^)^ and polyphenols^(^^26^^–^^28^^)^ and/or rich in both compounds^(^^29^^)^ have an impact on the gut microbiota, including cocoa^(^^26^^)^, pomegranate^(^^14^^)^, wine^(^^28^^)^, blueberries^(^^27^^)^, whole-grain cereal^(^^18^^)^, maize-derived whole grain^(^^22^^)^, artichoke^(^^19^^,^^20^^)^ and apples^(^^29^^)^. The effects of such foods are likely to be dependent on both the dietary fibres and polyphenols that they contain, and it has been suggested that such foods may be an effective strategy for the maintenance of a beneficial gut microbial ecology, leading to better gastrointestinal health^(^^30^^)^.

Date fruit (*Phoenix dactylifera* L.) contains high levels of both dietary fibre and polyphenols. Although an important food in the Middle East and North Africa, dates are also cultivated and consumed to an increasing degree in some parts of the USA, in particular Southern California, Arizona and Texas^(^^31^^)^. Dates are rich in energy and carbohydrates, mainly fructose, glucose and sucrose, which are absorbed in the upper gut, but also contain relatively high amounts of dietary fibres (6·4–11·5 %). The latter exist mainly as insoluble fibre with smaller amounts of soluble fibre^(^^32^^)^. Previously, we have shown that dates also contain significant amounts of polyphenols including phenolic acids (gallic, protocatechuic, hydroxybenzoic, vanillic, isovanillic, syringic, caffeic, ferulic, sinapic, *p*-coumaric, isoferulic), flavonoid glycosides (quercetin, luteolin, apigenin and kaempferol) and anthocyanidins^(^^33^^)^. Furthermore, polyphenol levels were observed to vary depending on cultivar type and degree of ripening: *kimri* (unripe), *khalal* (full-size, crunchy), *rutab* (ripe, soft) and the final *tamr* stage (ripe, reduced moisture)^(^^34^^)^. The Ajwa variety of date, a common cultivar grown in Saudi Arabia, has been observed previously to exhibit anti-inflammatory and antioxidant potentials^(^^35^^)^. In our previous work, Ajwa dates were considered to be the richest in polyphenols, specifically anthocyanidins, in comparison with other varieties, such as Barni and Khalas; however, polyphenols content reduce dramatically at the last stage of ripening^(^^33^^)^. As well as investigating the impact of the whole date fruit on gut microbiology, the present study aimed to assess the potential of these polyphenols to exert effects on the gut microbiota. Polyphenols are usually poorly absorbed from the small intestine and hence reach the colon where they are subject to extensive biodegradation by the resident microbiota^(^^36^^,^^37^^)^, although the effects of this metabolism on the growth of the microbiota is less well understood. Furthermore, date polyphenols and their metabolites, generated by interaction with the gut bacteria, may also favour the colonic epithelium, via their potential to inhibit proliferation of human colon cancer cells. The present study was designed to assess the impact of whole date fruit and date polyphenol extracts (DPE) on the faecal microbiota, using pH-controlled, mixed faecal batch cultures. In addition, bacterial metabolites, such as SCFA and phenolic metabolites, were also measured. The secondary aim was to assess the potential of both whole date and DPE to inhibit colon cancer cell growth using Caco-2 cells.

## Materials and methods

### Collection of dates

Ajwa date fruit were harvested at the *tamr* stage from Al-Gudaibi farm (Al-Qaseem, Saudi Arabia) 1 week before transportation to the UK. Date fruits were transported to the UK in polyethylene boxes at 4°C. Samples were then stored at –20°C before extraction and digestion.

### Preparation of extracts

#### Date polyphenol extracts

Dates (100 g) were pitted, weighed and homogenised in 300 ml methanol–water (4:1; v/v) containing 10 % NaF (1 m) to inhibit polyphenol oxidase^(^^31^^)^. Extracts were stirred for 2 h at 20°C and then filtered through a sintered funnel (porosity = 1) to remove solids. Aqueous methanol extracts were concentrated under vacuum using a rotator vacuum evaporator (ORME Scientific Ltd) and the remaining residue was diluted in acidified water (pH 2; HCl). Sugars were removed by adding 3 g of the extract to a XAD-16 resin packed column (50 cm length х 2·2 cm diameter) (Sigma Aldrich)^(^^38^^)^. Elution of sugars was achieved by the addition of 100 ml of acidified water (pH 2; HCl), followed by 300 ml of distilled water at a constant rate (0·5 ml/min) using a diaphragm-metering pump (STEPDOS; Scientific Laboratory Suppliers). The Fehling test was carried out to ascertain the presence of sugars in the water extracts^(^^39^^)^. Following removal of all sugars, elution of phenolics was achieved by the addition of 400 ml of methanol at a flow rate of 0·5 ml/min. The eluent collected was concentrated using a rotator vacuum evaporator at 40°C and the concentrated extract stored at –80°C until analysis. The 100 g of dates contained 1500 mg of polyphenols by weight. The polyphenol profile has been characterised in prior experiments, which was published in our previous study^(^^33^^)^.

#### Digested date extracts

Date fruits were pitted and 60 g of sample were added to 150 ml distilled water and mixed in a stomacher for 2 min. The solution was then mixed with α-amylase (20 mg) in CaCl_2_ (1 mm; 6·25 ml) and incubated at 37°C for 30 min on a shaker, which is called the oral digestion phase. In the following gastric phase of digestion, pepsin (2·7 g) was dissolved in HCl (0·1 m; 25 ml) and then the mixed sample was added. The pH was adjusted to 2 using HCl (6 m) and incubated at 37°C for 2 h on a shaker. The last digestion was the small-intestinal phase where pancreatin (560 mg) and bile (3·5 g) were dissolved in NaHCO_3_ (125 ml) and the sample added. pH was adjusted to 7 using NaOH (6 m) and incubated at 37°C for 3 h on a shaker. Samples were transferred to cellulose dialysis membranes (1 kDa molecular weight), purchased from Cheshire Biotech, to be dialysed against NaCl (0·01 m; 5°C) to remove low-molecular mass digestion products. After 15 h, the dialysis fluid was changed and dialysis continued for an additional 2 h. Afterwards, samples were freeze dried (5 d) and were ready to be used in *in vitro* fermentation. All chemicals were purchased from Sigma Aldrich.

### Faecal sample preparation

Faecal samples were obtained from three healthy volunteers, who had not consumed any antibiotics for at least 6 months before the study and had no history of gastrointestinal disease. Volunteers were not regular consumers of probiotic/prebiotic supplements. Samples were prepared on the day of the experiment and within 1 h of production were diluted (1:10, w/v) in an anaerobic phosphate buffer (0·1 m; pH 7·4). Faecal samples were then homogenised in a stomacher for 2 min, then sieved, forming faecal slurries from each volunteer used in three different batch culture experiments.

### Preparation of fermentation vessels

Basal nutrient medium was prepared before the experiment, by adding peptone water (2 g/l), yeast extract (2 g/l), NaCl (0·1 g/l), K_2_HPO_4_ (0·04 g/l), KH_2_PO_4_ (0·04 g/l), NaHCO_3_ (2 g/l), MgSO_4_.7H_2_O (0·01 g/l), CaCl_2_.6H_2_O (0·01 g/l), Tween 80 (2 ml/l), haemin (50 mg/l), vitamin K_1_ (10 ml/l), l-cysteine (0·5 g/l), bile salts (0·5 g/l), resazurin (1 mg/l) and distilled water. The autoclaved medium was aseptically added to the batch-culture vessels. Vessels were sparged overnight with O_2_-free N_2_ at a rate of 15 ml/min. Before inoculation, pH of the medium was adjusted to 6·8, using both basic (1 m-NaOH) and acidic (1 m-HCl) solutions, which were monitored and modulated by a pH controller.

### Inoculation of substrate in the batch culture

At the start of the experiment, 15 ml of faecal slurry (1:10, w/v) and substrates were added to each batch-culture vessel. The first experiment consisted of three vessels: (1), containing the DPE (1·5 g of date fruit contained 150 mg/ml of polyphenols and other small molecular weight components extracted by XAD-column chromatography; these are expected to reach the human colon); (2), fructo-oligosaccharide (FOS) (1 %, w/v, 1·5 g Raftilose P95) purchased from Orafti (1 %, w/v); and (3), a control vessel (without a substrate). For the second experiment, three vessels were run: (1), containing digested date extract (DDE) (1·5 g, containing polyphenols and dietary fibres); (2), FOS (1 %, w/v, 1·5 g Raftilose P95); and (3), a control vessel (without a substrate). The concentrations chosen in this experiment reflect the amounts that would reach the colon after consumption (1·5 g containing 150 mg of polyphenols in the culture model is approximately equivalent to 100 g of dates giving 1500 mg/1500 ml, which is consuming about seven to ten pieces of date fruit). Both experiments (batch cultures were carried out in triplicates) were run under anaerobic conditions for 48 h and 7 ml of sample collected at five time points (0, 5, 10, 24 and 48 h) analysed for bacterial count using fluorescence *in situ* hybridisation (FISH) analysis, metabolites using HPLC and cancer cell inhibition using the sulforhodamine B (SRB) assay.

### Bacterial enumeration using fluorescence *in situ* hybridisation

This method has been described by Daims *et al*.^(^^40^^)^ where fermented samples were prepared, fixed and hybridised to be ready for counting. Batch-cultured samples were removed at different time points (0, 5, 10 and 24 h) and suspended to concentrate the bacterial cells. Fixation took place by adding the batch cultures in 4 % (w/v) paraformaldehyde. Samples were incubated for 3 to 12 h at 4°C without freezing, and then centrifuged at 15 000 ***g*** for 5 min to remove residual paraformaldehyde. Cells were resuspended in PBS–ethanol and stored at –20°C until FISH analysis. For FISH analysis, samples were diluted with PBS/SDS (sodium dodecyl sulphate) diluents and applied to six-well slides for hybridisation. Dilutions were chosen, according to the each fluorescent-labelled 16S rRNA-targeted oligonucleotide probe. These probes are labelled with the fluorescent dye Cy3 to enumerate bacterial cells in samples. FISH analysis was conducted using six different probes: Bif164 for *Bifidobacterium*^(^^41^^)^; Lab158 for *Lactobacillus–Enterococcus*^(^^42^^)^; ATO for *Atopobium–Coriobacterium* group^(^^43^^)^; Erec482 for *Clostridium coccoides*–*Eubacterium rectale*^(^^44^^)^; Chis150 for *Clostridium* subgrp. *histolyticum*^(^^45^^)^; Bac303 for *Bacteroides*–*Prevotella*^(^^42^^)^; and EUB338 for total bacteria^(^^46^^)^. Then 20 µl of the diluted samples were added to each well of the six-well slides. Lysosyme was added with certain types of probes, such as, Lab158, to ensure sufficient permeability of the cell envelope, and allowing the probes to enter cells efficiently. Slides were then dehydrated in 50, 80 and 96 %, respectively, and hybridisation mix was added to the wells. Hybridisation was set to the appropriate temperature, according to the type of probe used. Once the hybridisation mix was added to all the wells, slides were added in an oven for 4 h. Slides were removed after the incubation period, and placed in a washing solution to remove any residuals. Then 20 µl of DAPI (4′,6′-diamino-2-phenylindole) were added to the washing solutions, which allowed the cells in the samples to be easily found under the microscope. Antifade (5 µl; Sigma Aldrich) was added to all the wells when slides were dried and a coverslip was placed on them to be ready for microscopic enumeration. Slides were stored in an opaque closed box in the fridge. The number of bacterial cells was counted using fluorescence microscopy (Nikon Eclipse E400; Nikon), which had an appropriate filter for the DAPI stain (excited at 359 nm and emits at 461 nm) and Cy3 dye (excited at 550 nm and emits at 565 nm). Fifteen to twenty fields were counted for each well in each six-well slide (Tekdon Inc.).

### Identification and quantification of bacterial metabolites

#### Phenolic compounds

DDE were centrifuged at 13 000 ***g*** for 5 min to remove all particulate matter and filtered through 0·45 µm acrodisc filters before injection (50 µl) onto the HPLC system. HPLC analysis was performed using an Agilent 1100 Series linked to diode array detector. Separation of compounds was achieved using a C18 Nova Pak® column (250 mm × 4·6 mm internal diameter, 5 µm particle size), fitted with a guard column (C18 NovaPak®; Waters Ltd). The mobile phase consisted of A: 5 m-hydrochloric acid (0·1 %) in 5 % aqueous methanol; and B: 5 m-hydrochloric acid (0·1 %) in aqueous acetonitrile (1:1) and was pumped through the column at 0·7 ml/min. Samples (50 µl) were injected and separated using the following gradient system (min/% B): 0/5, 5/5, 40/50, 55/100, 59·9/100 and 60/5 for the detection of all compounds. The eluent was monitored by photodiode array detection at 254, 280, 320, 370 and 520 nm and spectra of products obtained over the 220–600 nm range. Phenolic compounds were characterised by their retention time and by comparison with known phenolic standards (0–100 µm; *R* > 0·995). All data were analysed using ChemStation® software. For identification of phenolic compounds, we used different standards, such as phenolic compound standards including gallic, protocatechuic, *p*-hydroxybenzoic, vanillic, isovanillic, caffeic, syringic, *p*-coumaric, ferulic, isoferulic and sinapic acids, (+)-catechin, (−)-epicatechin, rutin, myricetin, quercetin, luteolin, naringenin, kaempferol, isorhamnetin, apigenin and petunidin (Sigma Aldrich).

#### Liquid chromatography–electrospray ionisation–MS/MS analysis

Methanol extracts were also analysed by liquid chromatography–MS/MS utilising electrospray ionisation. This consisted of an Agilent 1200 HPLC system equipped with a binary pump, degasser, autosampler, thermostat, column heater; photodiode array detector and an Agilent 1100 Series LC/MSD mass trap spectrometer. Separation of samples was achieved using a Zorbax SB C18 column (2·1 × 100 mm; 1·8 µm; Agilent) and HPLC conditions were as follows: injection volume, 5 µl; column temperature, 25°C; binary mobile system, (A) 0·1 % aqueous formic acid and (B) 0·1 % of formic acid in acetonitrile; flow rate, 0·2 ml/min. A series of linear gradients were used for separation (min/%B): 0/10, 3/10, 15/40, 40/70, 50/70 and 65/10. MS was performed in the negative ion mode (scan range, *m/z* 100–800 Da; source temperature, 350°C). All solvents used were of liquid chromatography–MS grade.

#### SCFA

DDE were centrifuged at 13 000 ***g*** for 5 min to remove all particulate matter and supernatant fractions were filtered through 0·2 µm acrodisc filters before injection (20 µl) onto the HPLC system (MERCK) equipped with refractive index (RI) detection. Separation of compounds was achieved using an ion-exclusion REZEX-ROA Organic acid column (Phenomenex) maintained at 85°C. Sulfuric acid in HPLC-grade water (0·0025 mmol/l) was used as an eluent and the flow rate was maintained at 0·5 ml/min. Quantification of the samples was obtained through calibration curves of lactic, acetic, propionic, butyric and valeric acids in concentrations between 12·5 and 100 mm.

### Caco-2 cancer growth

DDE and DPE were tested for the percentage growth inhibition of Caco-2 cells before and after pH-controlled batch culture fermentation at 0 and 10 h, relative to untreated cells. Samples were centrifuged at 13 000 ***g*** for 10 min to remove all particulate matter and supernatant fractions were filtered through 0·22 µm acrodisc filters. Caco-2 cells European Collection of Cell Cultures (*ECACC*) (http://www.ecacc.org/)) were cultured in Dulbecco's modified Eagle's medium, supplemented with 20 % heat-inactivated bovine serum, 2 mm-l-glutamine, 1 % non-essential amino acids, 100 U/ml penicillin and 100 µg/ml streptomycin (PAA Cell Culture Company). Anti-proliferative ability was assessed using the SRB assay. Cells were seeded in twenty-four-well plates at low confluence (5 × 10^4^ per well) and exposed to all extracts (0·2 mg/ml). Cells were harvested following 24, 48 and 72 h in culture and fixed by the addition of 125 µl ice-cold TCA (10 % final concentration; 4°C; 1 h). After fixing, the medium was removed, cells were washed and total biomass determined using SRB (500 µl of 0·4 % SRB; 0·5 h) (Sigma Aldrich). Unincorporated dye was removed by washing with 1 % acetic acid, whilst cell incorporated dye was solubilised using Tris–base (10 mm). Dye incorporation, reflecting cell biomass, was measured at 492 nm, using a GENios microplate reader (TECAN).

### Statistical analysis

DDE and DPE were tested in two different pH-controlled batch-culture experiments, using three different donors in three separate experiments. Changes in both bacterial counts (log_10_) and SCFA (mm) were expressed relative to the control and standard deviation. Changes in the percentage growth inhibition in Caco-2 cells were measured relative to untreated cells. One-way ANOVA was applied to show significant differences among different time points of fermentation (0, 5, 10, 24 and 48 h). Significant differences between times points were detected using least significant difference (LSD) tests. SPSS software, version 18·0, was used (IBM).

Dietary fibre were analysed by Campden BRI Laboratories (AOAC method 991·43); Ajwa total fibre content is 6·85–7·9 g/100 g (6·15–7·2 g insoluble fibre).

## Results

### Effects of digested date extract and date polyphenol extract in specific bacterial groups

Selected bacterial groups were assessed by FISH following faecal batch-culture fermentation experiments using DDE and DPE. Results indicated that both were capable of inducing significant modulation in the growth of specific bacterial groups at different time points (Tables 1 and 2). DDE significantly increased bifidobacteria (*P* < 0·05) at 5 and 10 h, and also a change in bacteroides at 24 h (*P* < 0·05), along with changes in total bacterial counts (*P* < 0·05) (Table 1). In contrast, DPE significantly increased bifidobacteria at 5 h (*P* < 0·05), whereas a decrease in bacteroides at 48 h (*P* < 0·05) was seen. Overall, DPE exhibited a weaker impact on bifidobacteria counts. In comparison with the prebiotic FOS, even though both DDE and DPE exhibited a bifidogenic effect, it was significantly smaller than FOS at different time points. DDE was much closer to FOS in its impact on bacterial composition change than DPE, where polyphenols have been shown to reduce bacteroides counts.

**Table 1.** Faecal bacterial numbers in three pH-controlled batch cultures over 48 h periods† (Mean values and standard deviations)

Probe…	Bif164		Lab158		Ato291		Bac303		Erec482		Chis150		Eub I-II-III	
	Mean	sd	Mean	sd	Mean	sd	Mean	sd	Mean	sd	Mean	sd	Mean	sd
FOS (0 h)	7·88	0·30	6·01	0·79	7·50	0·11	8·21	0·07	8·44	0·01	5·56	0·01	9·18	0·24
FOS (5 h)	8·09*	0·33	6·69*	0·45	7·69	1·04	8·12	0·19	8·67	0·37	6·15	0·08	9·00*	0·16
FOS (10 h)	8·53**	0·21	6·13	0·98	7·70	1·20	8·06	0·46	8·81	0·41	5·76	0·00	9·38*	0·11
FOS (24 h)	8·34*	0·16	5·98	0·74	7·72	0·80	8·23	0·17	8·66	0·39	5·55	0·02	9·27	0·05
FOS (48 h)	8·47*	0·05	6·07	0·57	7·74	0·98	8·08	0·21	8·82	0·36	5·55	0·02	9·44**	0·14
DDE (0 h)	7·83	0·36	6·11	0·10	7·28	0·49	8·09	0·43	8·27	0·23	5·56	0·01	9·06	0·20
DDE (5 h)	8·24*	0·20	6·15	0·75	7·59	0·97	7·73	0·23	7·91	0·66	5·56	0·01	8·92	0·34
DDE (10 h)	8·21*	0·20	6·25	0·86	7·71	1·16	7·90	0·44	8·56	0·46	5·56	0·01	9·15	0·26
DDE (24 h)	8·22	0·32	5·75	0·59	7·86	0·80	8·20*	0·07	8·66	0·28	5·55	0·02	9·40*	0·03
DDE (48 h)	8·28	0·53	6·19	0·74	7·81	0·74	7·96	0·20	8·54	0·53	5·55	0·02	9·41*	0·12

FOS, fructo-oligosaccharides; DDE, digested date extract.

Mean value was significantly different from that at 0 h fermentation: *P < 0·05, **P < 0·01 (one-way ANOVA and least significant difference (LSD) test).

† Bacterial counts in fermented faecal samples were determined by fluorescence *in situ* hybridisation and are expressed as log_10_ cells/g faeces.

**Table 2.** Faecal bacterial numbers in three pH-controlled batch cultures over 48 h periods† (Mean values and standard deviations)

Probe…	Bif164		Lab158		Ato291		Bac303		Erec482		Chis150		Eub I-II-III	
	Mean	sd	Mean	sd	Mean	sd	Mean	sd	Mean	sd	Mean	sd	Mean	sd
FOS (0 h)	8·34	0·23	6·06	0·49	7·75	0·09	8·33	0·17	8·18	0·23	5·59	0·07	8·96	0·05
FOS (5 h)	8·53	0·31	6·55	0·60	7·90	0·08	8·08	0·38	7·82	0·43	5·79	0·53	9·05	0·24
FOS (10 h)	8·55	0·31	6·86*	0·43	8·23*	0·08	8·18	0·36	8·11	0·24	6·21	0·72	9·20*	0·03
FOS (24 h)	8·55	0·26	7·04*	0·42	7·72	0·30	7·99	0·36	8·25	0·08	5·47	0·15	9·43**	0·03
FOS (48 h)	8·69*	0·16	7·02*	0·49	8·16*	0·04	7·90	0·34	7·99	0·29	5·53	0·17	9·33	0·19
DPE (0 h)	8·13	0·10	5·98	0·61	7·95	0·27	8·27	0·22	8·29	0·18	5·85	0·63	9·11	0·11
DPE (5 h)	8·24*	0·15	6·22	0·47	7·70	0·44	7·94	0·14	8·03	0·18	5·77	0·38	9·15	0·19
DPE (10 h)	8·20	0·13	6·35	0·42	7·77	0·18	8·03	0·12	7·97	0·21	5·84	0·39	9·24	0·05
DPE (24 h)	8·32	0·15	6·34	0·63	7·71	0·37	7·94	0·26	8·03	0·26	5·60	0·32	9·26	0·12
DPE (48 h)	8·18	0·24	6·42	0·62	7·54	0·21	7·83*	0·31	7·86	0·08	5·53	0·17	9·24	0·18

FOS, fructo-oligosaccharides; DPE, date polyphenol extract.

Mean value was significantly different from that at 0 h fermentation: *P < 0·05, **P < 0·01 (one-way ANOVA and least significant difference (LSD) test).

† Bacterial counts in fermented faecal samples were determined by fluorescence *in situ* hybridisation and are expressed as log_10_ cells/g faeces.

### Changes in bacterial metabolites

To investigate how different bioactive compounds in date fruits (polyphenols and fibres) were metabolised by the faecal microbiota, we utilised HPLC analysis to analyse SCFA after DDE fermentation and polyphenols after DPE fermentation. With regards to DDE fermentation, there were increases in acetate, propionate, butyrate and lactate among time points in comparison with 0 h. However, a significant increase (*P* < 0·05) was only observed with acetate concentrations at 48 h fermentation (Table 3). With regards to DPE fermentation, there were significant reductions in luteolin at 10 h (*P* < 0·05), 24 h and 48 h and quercetin (*P* < 0·01) concentrations in comparison with 0 h whereas apigenin and petunidin were significantly reduced (*P* < 0·05) at 5 h fermentation (Table 4). The current batch culture confirms the presence of petunidin at a higher level, in comparison with other aglycones as seen in previous work^(^^33^^)^.

**Table 3.** SCFA concentrations in three pH-controlled batch cultures over 48 h periods† (Mean values and standard deviations)

Time…	0 h		5 h		10 h		24 h		48 h	
	Mean	sd	Mean	sd	Mean	sd	Mean	sd	Mean	sd
Lactate (mm)	2·10	3·64	6·17	10·69	7·94	13·75	11·47	12·06	6·24	5·47
Formate (mm)	9·44	10·44	14·46	11·93	5·73	5·54	5·18	5·00	5·16	5·00
Acetate (mm)	13·26	12·47	25·01	17·73	22·96	17·89	45·77	13·60	45·35*	1·40
Propionate (mm)	6·38	1·69	9·07	2·34	12·82	10·79	11·64	4·86	9·97	3·76
Butyrate (mm)	3·99	3·5	4·98	2·30	5·15	5·81	7·51	5·31	10·41	2·99
Valerate (mm)	1·44	2·49	1·51	2·59	1·66	2·88	1·03	1·78	0·49	0·86

* Mean value was significantly different from that at 0 h fermentation (*P* < 0·05; ANOVA and least significant difference (LSD) test).

† Metabolite counts in fermented faecal samples were determined by HPLC.

**Table 4.** Aglycone concentrations in three pH-controlled batch cultures over 48 h periods‡ (Mean values and standard deviations)

Time…	0 h		5 h		10 h		24 h		48 h	
	Mean	sd	Mean	sd	Mean	sd	Mean	sd	Mean	sd
Myricetin (mm)	0·21	0·07	0·19	0·06	0·25	0·05	0·21	0·06	0·21	0·03
Luteolin (mm)	0·32	0·04	0·16†	0·02	0·15*	0·02	0·00**	0·00	0·00**	0·00
Quercetin (mm)	0·30	0·03	0·18†	0·02	0·18	0·07	0·00**	0·00	0·00**	0·00
Apigenin (mm)	0·00	0·00	0·31*	0·12	0·14	0·29	0·00	0·00	0·00	0·00
Petunidin (mm)	0·95	0·29	0·37*	0·11	0·43	0·19	0·33	0·185	0·30	0·19

* Mean value was significantly different from that at 0 h fermentation: *P < 0·05, **P < 0·01 (one-way ANOVA and least significant difference (LSD) test).

† Mean value was borderline significantly different from that at 0 h fermentation (*P* > 0·05; one-way ANOVA and least significant difference (LSD) test).

‡ Polyphenol compound counts in fermented faecal samples were determined by HPLC.

### Caco-2 cell proliferation

**Fig. 1.**
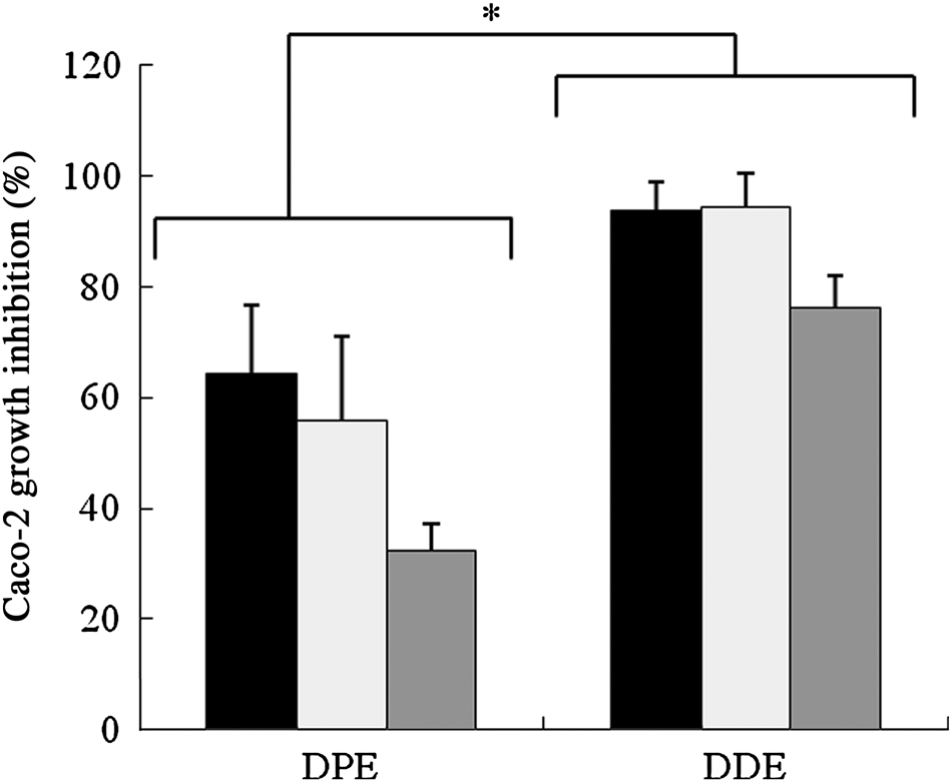
Caco-2 growth inhibition percentages measured in pH-controlled batch cultures over 48 h periods: ■, before fermentation; □, 10 h fermentation; 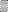, 48 h fermentation. Percentages were measured by spectrophotometer at 570 nm. Values are means, with standard deviations represented by vertical bars. * Mean values for date polyphenol extract (DPE) were significantly different from those for digested date extract (DDE) (*P* < 0·05; one-way ANOVA and least significant difference (LSD) test).

We also assessed the ability of DDE, DPE and metabolites generated by fermentation to inhibit colon cancer cell proliferation (Fig. 1). Here, both extracts induced significant antiproliferative action before and following bacterial fermentation. Specifically, DDE had a significantly (*P* < 0·05) greater ability to reduce the growth of Caco-2 cells than DPE, inducing about 90 % of inhibition at 48 h of exposure. Fermentation was observed to decrease overall cancer cell growth inhibition, only achieving a 30 % inhibition with DPE and 70 % inhibition with DDE after 48 h fermentation.

## Discussion

The present study has provided evidence that both DDE and DPE derived from Ajwa dates are able to significantly increase the growth of beneficial bacteria, such as bifidobacteria in human faecal batch cultures. Such changes in the growth of these bacteria may play a role in enhancing colon health, by inhibiting the growth of pathogens and increasing the production of acetate and lactate^(^^47^^)^. Previous studies have indicated that malvidin-3-glycoside (200 mg/l) may induce significant increases in bifidobacteria, lactobacilli and total bacteria^(^^15^^)^, fitting well with our observation in that the Ajwa dates used are, unlike other dates, rich in anthocyanins^(^^33^^)^. With regards to other bacterial groups tested, lactobacilli and *C. histolyticum* did not change with either of the treatments. Such data indicate a selective effect on the growth of specific bacteria, something that is commonly seen with prebiotics such as FOS^(^^15^^)^. The number of bacteroides, on the other hand, was significantly increased at 24 h of exposure to DDE, but was decreased when exposed to DPE. Bacteroides are considered a dominant bacterial group in the large intestine and are known to produce propionate when they ferment dietary fibres from oats and barley^(^^8^^)^. In previous studies, limited changes have been found with bacteroides after polyphenol- and/or fibre-rich foods. In the present study, the decrease in bacteroides seen with DPE fermentation may be due to the use of pure polyphenol extracts, where polyphenols exhibit the ability to bind to bacterial cells membranes, thus inhibiting their growth. Differences between polyphenols' antimicrobial activities depend also on the bacterial lipid bilayer, which may have a greater affinity to inhibit Gram-negative bacteria, such as bacteroides^(^^48^^)^. On the other hand, with DDE, the presence of fibres, and cleaved sugars from polyphenol glycosides should interfere with the selectivity of bacterial growth, where bacteroides and total bacteria were seen to be increased.

The whole Ajwa dates (DDE) were also observed to induce an increased trend in the *Atopobium–Coriobacterium* group at all time points, which is something previously observed with oat and barley fermentation, containing similar amounts of insoluble fibres^(^^8^^)^. *Atopobium* spp. have recently been reported as being capable of modulating caspase-9 and caspase-3 in a manner known to induce apoptosis and inhibiting Caco-2 cancer growth *in vitro*^(^^49^^)^. In addition, an increased trend in the growth of *C. coccoides–E. rectale* with DDE was also observed. This bacterial group is believed to produce butyrate, which is considered protective with respect to effects on colon cancer^(^^50^^)^ and ulcerative colitis^(^^51^^)^. Furthermore, some species of the *Coccoides–Eubacterium* group are thought to reduce cancer risks due to butyrate production^(^^52^^)^, again through an inhibition of apoptosis^(^^53^^)^. Previously, most interest has focused on the potential of established prebiotics to increase the growth of bifidobacteria and lactobacilli, with little interest in the growth of other types of bacteria such as *Atopobium* spp., *E. rectale* spp. and *Roseburia* that may be capable of exerting anti-cancer abilities^(^^49^^)^. Costabile *et al.* revealed an increase in *Atopobium* spp. following the consumption of inulin extracted from artichokes in human subjects^(^^19^^)^, and butyrate-producing bacteria via polydextrose consumption^(^^54^^)^. Therefore, our findings must be tested in a larger number of volunteers to ascertain such changes.

The present study shows a bifidogenic effect with both whole date extracts and polyphenol extracts^(^^6^^,^^11^^)^, where a possible potential is worth testing in human subjects. The effects of polyphenols on the growth of the gut microbiota appear to depend on their structure. For example, no changes in the growth of lactobacilli or bacteroides were observed after exposure to catechins^(^^12^^)^ and proanthocyanidins^(^^16^^)^, whereas with wine phenolic extracts^(^^13^^)^, and flavanol-3-ols present in grape seed, significant increases were observed^(^^17^^)^. Rather, our data show that there was a selective shift in the bacterial population of the large intestine, with whole date fruit (DDE), but still considered a weak bifidogenic impact when compared with cereals^(^^6^^,^^8^^)^. A fraction of such impact should be due to the presence of polyphenols, where bifidobacteria have been seen to be significantly increased via inoculation. Questions remain as to the extent to which polyphenols have the ability to modulate the gut microbiota towards a healthier state, with PCR, DNA sequencing, metabonomics and metabolomics required to fully understand the mechanisms involved in polyphenol fermentation^(^^55^^)^ and whether there may be synergetic actions of both polyphenols and dietary fibres on bacterial growth in the large gut. In order to exert such effects in the large intestine, date polyphenols and fibres must escape metabolism in the upper gastrointestinal tract, thus reaching the colon intact where they may exhibit functional properties within. Previous studies have suggested that up to 80 % of polyphenols similar to those identified in dates^(^^33^^)^ may reach the colon^(^^37^^)^, where they are then hydrolysed by bacteria and further metabolised to smaller molecular-weight phenolic endproducts^(^^36^^,^^56^^)^. According to Tzounis *et al*.^(^^12^^)^, HPLC chromatograms showed that 50 % of flavanol monomers are metabolised by the gut bacteria, with (+)-catechin being converted to (+)-epicatchein. Date fruits are known to contain high amounts of phenolic acids, procyanidins and flavonoid glycosides^(^^31^^,^^33^^)^. In the present study, myrecitin, luteolin, quercetin and apigenin and the anthocyanidin petunidin were all detected, presumably following cleavage of their corresponding glycoside to the aglycone. Previously, it has been suggested that flavonoid glycosides and anthocyanins undergo rapid hydrolysis by various gut bacteria, such as *E. ramulus*, to produce aglycones and other endproducts via the actions of microbial glycosidase leading to flavonoid ring fission^(^^48^^)^, and/or the action of *Bifidobacterium lactis*^(^^57^^)^. The more rapid metabolism of polyphenols in the DPE, relative to the whole date, is expected to be due to matrix effects, with polyphenols needing initial release in the whole fruit before any bacterial metabolism can take place^(^^15^^)^. In the present data, we observed the rapid formation of aglycones, probably as a result of specific bacterial enzymes that cleave the 3-glycosidic linkage, and the later production of phenolic acids, such as syringic acids, *p*-coumaric acids and gallic acids, as seen with malvidin glycosides in batch cultures^(^^58^^)^. In another study, peonidin-3-glycoside and cyanidin-3-glycoside degradation by the gut mircobiota resulted in the production of vanillic acids and protocatechuic acids^(^^59^^)^. Such compounds have been seen in previous work to induce apoptosis in colon cancer cell lines and interfere with the cellular signalling^(^^60^^)^.

SCFA are also produced as a result of saccharolytic metabolism of gut microbiota in the large intestine^(^^4^^)^. HPLC analysis was used to identify and quantify the main SCFA produced in batch cultures, indicating a significant increase in acetate concentrations, seen at 48 h, which was mostly associated with the increase in bifidobacteria numbers. Previous investigations have demonstrated a direct relationship between changes in bifidobacteria and acetate when pomegranate by-products were inoculated in batch cultures, whereas when punicalagins (pomegranate ellagitannins) were added no effects were detected in bacteria or in SCFA production, which indicates that modifications in the gut ecology were mostly due to other bioactive compounds, such as insoluble fibres^(^^14^^)^. The extent of degradation and metabolism of dietary components by bacterial enzymes depends on the different composition of bacteria within different volunteers, something that will contribute to the bioavailability and production of metabolic endproducts. In the present study, the increase in butyrate was not significant, which is mainly associated with butyrate-producing bacteria, *C. coccoides–E. rectale* that was not significantly affected following their exposure to DDE. The explanation behind the relatively slow production of SCFA following exposure to whole date fruit could again be the complex matrix^(^^48^^)^. In addition, donor diets may play crucial roles in colon metabolism, where the presence of other foods ingredients, such as proteins, could be involved in bacterial fermentation. As a result of bacterial hydrolysis, other by-products, such as bile acids and ammonia, could also interfere with the gut ecology^(^^61^^)^. SCFA such as butyrate are thought to be involved in colon cancer inhibition^(^^62^^)^. Changes in the colonic microflora, together with the production of metabolic products (SCFA and phenolic acids) may work together to exhibit an anti-cancer effect in the colon, thus enhancing gut health.

There is still debate as to whether foods rich in polyphenols and dietary fibres may prevent colon cancer in humans, as clinical data remain inconclusive^(^^63^^,^^64^^)^. However, the fact that we observed the potential of date polyphenols to induce apoptosis even after metabolism by the microbiota suggests that date intake may be capable of exerting anti-carcinogenic activity. Previously, polyphenols extracted from olive oil have also been shown to exert strong anti-proliferative effects in Caco-2 cell lines, along with changes in cellular signalling^(^^65^^,^^66^^)^. This anti-cancer effect was dose dependent and still apparent after bacterial metabolism of the original polyphenols in the extract. In the pure extracts, the presence of phenolic acids, flavonoid glycosides and anthocyanins contributed to a strong inhibition in Caco-2 cell lines, whereas with fermentation, inhibition was weaker due to the loss of phenolic acids and other possible unidentified compounds, which do not reach the colon. What was detected with the fermented extracts is more similar to the human dietary intake, which needs to be proved in human trials.

The present paper represents an early investigation into the influence of date fruit and date polyphenols on the growth of large-intestinal bacteria and related metabolites. The whole date fruit extract was capable of larger effects on bacterial growth than that seen with the date polyphenols alone, due to the presence of insoluble fibres, where polyphenols themselves showed a weaker ability to modify bacterial counts. Furthermore, these data were paralleled by changes in bacterial metabolic products; both SCFA and phenolic acids following incubation with the faecal microbiota were shown to inhibit colon cancer cell growth. The present results suggest that the synergistic action of both date polyphenols and insoluble fibres could enhance colonic health.
